# Effect of Ringer’s acetate in different doses on plasma volume in rat models of hypovolemia

**DOI:** 10.1186/s40635-017-0160-3

**Published:** 2017-10-26

**Authors:** Svajunas Statkevicius, Attila Frigyesi, Peter Bentzer

**Affiliations:** 1 Department of Anesthesia and Intensive Care, Skåne University Hospital, Lund, Sweden; 2 Department of Clinical Sciences, Lund University, Lund, Sweden; 3 Department of Anesthesia and Intensive Care, Helsingborg Hospital, Helsingborg, Sweden

**Keywords:** Plasma volume, Crystalloid, Dose–response, Inflammation, Sepsis, Hemorrhage

## Abstract

**Background:**

Even though crystalloids are the first choice for fluid resuscitation in hemodynamically unstable patients, their potency as plasma volume expanders in hypovolemia of different etiologies is largely unknown. The objective of the study was to investigate dose–response curves of a crystalloid in hypovolemia induced by either sepsis or hemorrhagic shock.

**Results:**

Rats were randomized to resuscitation with Ringers acetate at a dose 10, 30, 50, 75, or 100 ml/kg at 4 h after induction of sepsis by cecal ligation and puncture (CLP) or 2.5 h after a 30 ml/kg hemorrhage. Plasma volume (^125^I–albumin) was the primary outcome. Plasma volume decreased by about 11.8 (IQR 9.9–14.5) ml/kg relative baseline after CLP and increased dose-dependently by at most 5.8 (IQR 3.3–7.0) ml/kg in the 100 ml/kg group at 15 min after resuscitation. In the hemorrhage group, the plasma volume increased by at most 13.8 (IQR 7.1–15.0) ml/kg in 100 ml/kg group. Blood volumes at baseline, calculated using hematocrit and plasma volumes, were 72.4 (IQR 68.2–79.5) ml/kg in sepsis group and 71.1 (IQR 69.1–74.7) ml/kg in hemorrhage group. At 15 min after resuscitation with a dose of 100 ml/kg blood volumes increased to 54.8 (IQR 52.5–57.7) ml/kg and ; 49.6 (IQR 45.3–56.4) ml/kg, in the sepsis and hemorrhage groups, respectively. Plasma volume expansion as the percentage of dose at 15 min was 5.9 (IQR 2.5–8.8)% and 14.5 (IQR 12.1–20.0)% in the sepsis and hemorrhage groups, respectively. At 60 min, average plasma volume as the percentage of dose had decreased to 2.9 (IQR ([−2.9] − 8.3)% (*P* = 0.006) in the sepsis group whereas no change was detected in the hemorrhage group. A dose-dependent decrease in the plasma oncotic pressure, which was more marked in sepsis, was detected at 60 min after resuscitation.

**Conclusions:**

We conclude that the efficacy of Ringers acetate as a plasma volume expander is context dependent and that plasma volume expansion is lower than previously realized across a wide range of doses. Ringers acetate decreases plasma oncotic pressure in sepsis, in part, by mechanisms other than dilution.

## Background

Despite the fact that clinical guidelines recommend crystalloids for the initial fluid resuscitation in hemodynamically unstable patients with suspected hypovolemia, very little is known about the efficacy of crystalloids as plasma volume expanders at different doses [1, 2]. Crystalloids distribute primarily in the extracellular space and following equilibration only 20–25% is thought to remain in the circulation [3]. Data supporting this distribution is mainly based on experiments performed in models of an acute hemorrhage and on postoperative patients using only one volume (dose) of crystalloid [4–7]. In these settings, homeostatic mechanisms acting to replace the lost fluid are likely to contribute to the increase in plasma volume after resuscitation [5, 8]. This is in contrast to sepsis and other inflammatory conditions in which the disruption of homeostatic mechanisms striving to maintain normovolemia most likely contributes to hypovolemia [9–12]. Based on this, it could be hypothesized that crystalloids are less potent as plasma volume expanders during inflammatory conditions. Results showing that only 1–9% of a crystalloid remain intravascularly immediately after resuscitation in experimental sepsis [13, 14] or in postoperative cardiac surgery patients, align with this hypothesis [15]. These results raise the following questions: Can normovolemia be achieved with a crystalloid-based resuscitation strategy in severe inflammation? Is the efficacy of crystalloids as plasma volume expanders context dependent, i.e. different in inflammatory conditions of different origin?

The present study was designed to investigate the dose–response relationship of a crystalloid in inflammatory conditions of different etiology. For this purpose, we evaluated the effect of resuscitation with an isosmotic Ringers acetate solution in different doses on the plasma volume in rat models of abdominal sepsis or hemorrhagic shock.

## Methods

### Animals

The study was approved by the Lund University Ethical Committee for Animal Research (M309–12) and animals were treated under the guidelines of the National Institutes of Health for Care and Use of Laboratory Animals. A total of 127 adult male Sprague–Dawley rats weighing 351 ± 21 g were used in the study.

### Anesthesia and preparation

Anesthesia was induced by inhalation of 5% isoflurane. After a tracheostomy, the animals were connected to a ventilator and ventilated with humidified air with a tidal volume of 10 ml/kg and a positive end-expiratory pressure of 3–4 cm H_2_O. The core temperature was kept at 37.1–37.3 °C using a heating pad. The left femoral artery was cannulated for the measurement of mean arterial blood pressure (MAP) and pulse pressure (PP), and to obtain blood samples for measurement of blood gases, sodium, lactate and hematocrit (I-STAT, Abbot Park, Ill). PP variation (PPV)(%) during a ventilatory cycle was used as measure of fluid responsiveness and calculated as; [PPmax-PPmin/(PPmax + PPmin/2)]*100 and is expressed as the mean value for 5 consecutive ventilator cycles [16]. The right jugular and the left femoral veins were cannulated for injections and fluid administration. Following the start of a continuous 0.5 μg/kg/min fentanyl infusion, isoflurane concentration was lowered to 1.1–1.3%. Urine was collected in a vial placed at the external meatus of the urethra and the bladder was emptied by external compression after completion of preparation and at the end of the experiment. After completion of the protocol, the animals were killed with an intravenous injection of potassium chloride.

### Measurement of plasma volume

The plasma volume (PV) was estimated by a calculation of the volume of distribution for ^125^I–labeled human serum albumin (HSA) at 5 min following an injection of a known dose as described previously [17]. The volume of ^125^I–HSA (0.5 ml) was not included in the resuscitation volume. The amount of free ^125^I in the administered dose was measured after precipitation with 10% trichloroacetic acid and centrifugation and was found to be < 2.6% in all experiments. Samples were counted in a gamma counter and blood volumes were estimated by dividing plasma volumes by 1-Hct.

### Experimental protocol

The study consisted of 2 main groups: a sepsis group and a hemorrhage group. Following the preparation and the induction of sepsis or a hemorrhage as described in detail below, animals were randomized with regard to the dose of resuscitation fluid.

### Sepsis model

**Fig. 1:**
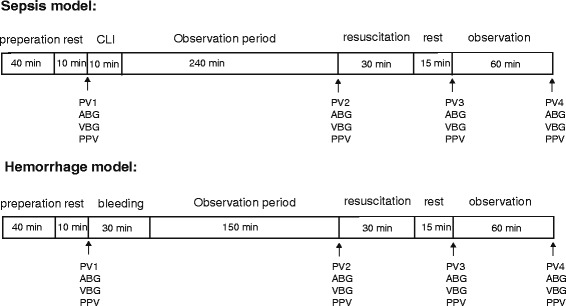
Schematic diagram of the experimental protocol in the hemorrhage and sepsis models. ABG (arterial blood gas), CLP (cecal ligation and puncture), PPV (pulse pressure variation), PV (plasma volume), VBG (venous blood gas)

Following baseline measurements (see Fig. 1), animals were subjected to a cecal ligation and puncture (CLP) procedure as described previously [14]. Four hours after the CLP procedure, plasma volume was measured again and the change in plasma volume was calculated. Animals with a plasma volume loss ≥ 5 ml/kg were included in the study and were randomized to treatment with either 0 ml/kg, 10 ml/kg, 30 ml/kg, 50 ml/kg, 75 ml/kg or 100 ml/kg of isosmotic Ringers acetate solution over a 30-min period (Plasmalyte®, Baxter). The plasma volume was measured again at 15 min and at 60 min after the completion of the fluid resuscitation.

### Hemorrhage model

After baseline measurements (see Fig. 1), animals were bled 30 ml/kg over 30 min via the cannulated femoral artery. At 2.5 h after the hemorrhage, the plasma volume was measured again and the change in plasma volume was calculated. The volume of bleeding and the duration of unresuscitated shock was chosen on the basis of a previous study demonstrating a shock induced inflammatory response during these condition [18]. Animals were then randomized to receive resuscitation with isosmotic Ringers acetate (Plasmalyte®, Baxter) at a dose of 0, 10, 30, 50, 75, or 100 ml/kg over a 30 min period. The plasma volume was measured at 15 and 60 min after completion of the fluid resuscitation.

### Plasma oncotic pressures and tissue water content

Oncotic pressure was measured using an osmometer with a 10-kDa cutoff membrane (Osmomat 050; Gonotec, Berlin, Germany) by an investigator blinded to the treatment status. To investigate the distribution of the resuscitation fluid, water content in the skin and subcutaneous tissue (from the abdominal wall), muscle (from the abdominal rectus muscle), lung, heart, liver, intestine and kidneys was measured. Tissue water content was also measured in two control groups of animals exposed to the CLP (*n* = 4) or the hemorrhage (*n* = 4) protocols and sacrificed before resuscitation. Tissues were extracted, weighted and dried for at least 72 h at 100 °C and then weighted again. Water content was calculated as (wet tissue weight – dry tissue weight)/wet tissue weight × 100.

### Statistics

Data was analyzed per protocol. Change in plasma volume at 15 min after resuscitation was the primary outcome. Because of the exploratory nature of these experiments, no formal power analysis was performed and the group size was based on previous studies in which differences in plasma volume expansion could be detected using the same methodology [13, 14]. Randomization was performed in blocks of four in each of the treatment groups with the objective to have eight to ten animals in each group. Animals that did not complete the protocol were replaced. Changes in plasma volume, hemodynamic parameters and laboratory data from the baseline to the end of the CLP or the hemorrhage procedure (before randomization) were evaluated using the paired Student’s *t* test. Differences in the plasma volume expansion and in hemodynamic and laboratory data within the sepsis and hemorrhage groups at the different time points after resuscitation were analyzed using one-way-ANOVA on ranks followed by an adjustment for multiple comparisons using the Dunn’s method. Differences in dose-dependent decrease in plasma oncotic pressure between the two groups were analyzed by comparing the slope of the dose–response curves as calculated by linear regression. Differences in tissue water content were analyzed using Mann–Whitney test. Data are presented as the median and interquartile range (IQR) unless stated otherwise.

## Results

### Mortality

**Fig. 2:**
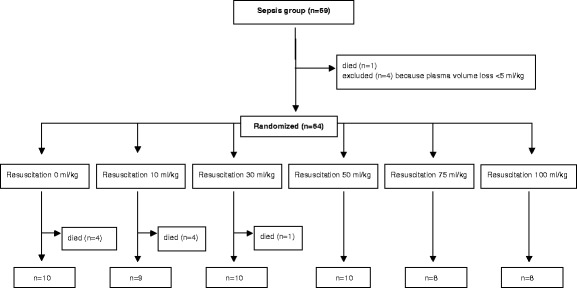
Flow chart of animals in the sepsis groups

**Fig. 3:**
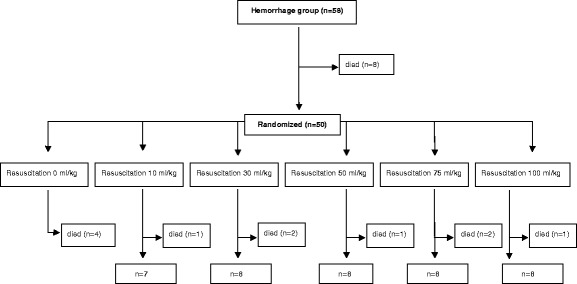
Flow chart of animals in the hemorrhage groups

The mortality in sepsis and hemorrhage was 14% (95% confidence interval [CI], 8–25%) and 33% (95% CI; 22–45%), respectively. A flow chart of the animals in both study arms is presented in Figs. 2 and 3.

### Hemodynamic and laboratory data

#### Sepsis

At the start of resuscitation, lactate, hematocrit and PPV had increased, and base excess, MAP and ScvO_2_ had decreased compared to the baseline values (*P* < 0.001 for all, see Table 1 for all hemodynamic and laboratory data). Both at 15 and at 60 min after resuscitation lactate decreased and base excess increased in the group receiving 100 ml/kg compared to the group receiving 10 ml/kg (*P* < 0.001 for both). The urine production was higher in animals resuscitated with a dose of 100 ml/kg compared to those resuscitated with a dose of 10 ml/kg (*P* = 0.022). Arterial pH and pCO_2_ did not differ between the groups at any of the time points (data not shown).

**Table 1:** Hemodynamic and laboratory data. Data are presented as median and IQR. The change in hemodynamic and laboratory data within the sepsis and hemorrhage groups at 15 and 60 min after resuscitation were analyzed using one-way ANOVA on ranks (* = *P* < 0.05 for comparisons with the 10 ml/kg group using the Dunn’s method to correct for multiple comparisons). MAP = mean arterial pressure, PPV = pulse pressure variation, ScvO2 = central venous saturation, Hct = hematocrit, BE = base excess, ∆ = change in respective parameter, ns = no survivors

	Sepsis						Hemorrhage					
Resus. volume ml/kg	0 (*n* = 10)	10 (*n* = 9)	30 (*n* = 10)	50 (*n* = 10)	75 (*n* = 8)	100 (*n* = 8)	0 (*n* = 4)	10 (*n* = 7)	30 (*n* = 8)	50 (*n* = 8)	75 (*n* = 8)	100 (*n* = 8)
Baseline												
MAP, mmHg	99 (73–106)	98 (89–105)	95 (86–109)	102 (81–117)	116 (93–121)	93 (79–115)	88 (71–106)	99 (85–105)	95 (84–106)	89 (78–104)	83 (65–98)	85 (76–94)
PPV, %	9 (7–11)	11 (7–15)	9 (7–13)	9 (9–13)	8 (6–9)	8 (7–15)	9 (4–16)	9 (7–11)	9 (7–11)	7 (4–11)	10 (7–11)	11 (7–13)
ScvO_2_, %	78 (71–80)	77 (73–80)	79 (76–82)	78 (71–84)	79 (66–81)	78 (75–83)	80 (75–83)	85 (83–88)	82 (79–85)	75 (73–79)	81 (71–84)	79 (74–83)
Lactate, mmol/L	2.1 (1.7–2.2)	2.2 (1.8–2.4)	2.1 (1.9–2.5)	2.3 (2.0–2.6)	2.0 (1.4–2.2)	2.1 (2.0–2.7)	1.9 (1.7–1.9)	2.4 (1.9–2.6)	2.0 (1.6–2.4)	2.2 (1.7–2.5)	2.5 (2.0–2.9)	2.1 (1.8–2.7)
Hct, %	42 (40–43)	41 (41–43)	42 (42–43)	43 (41–44)	43 (42–45)	42 (40–43)	43 (41–43)	44 (43–44)	42 (40–43)	42 (40–43)	41 (38–43)	42 (40–44)
BE, mEq/L	4 (3–5)	4 (3–4)	5 (3–5)	4 (3–4)	4 (3–5)	4 (3–5)	5 (4–7)	4 (3–5)	5 (4–6)	6 (3–6)	5 (4–6)	5 (3–6)
Pre- resuscitation												
MAP, mmHg	81 (67–92)	92 (87–108)	94 (85–98)	91 (76–96)	86 (67–100)	96 (81–109)	57 (51–66)	59 (50–80)	48 (45–51)	49 (40–55)	50 (47–51)	56 (53–60)
PPV, %	18 (15–21)	13 (12–22)	13 (12–19)	16 (15–19)	16 (13–21)	17 (15–18)	24 (19–27)	16 (12–19)	20 (10–32)	24 (13–28)	24 (19–28)	19 (17–27)
ScvO_2_, %	60 (50–70)	51 (39–57)	57 (53–59)	62 (45–69)	51 (45–68)	56 (50–61)	54 (46–61)	59 (51–69)	43 (40–53)	44 (33–52)	41 (37–48)	43 (32–50)
Lactate, mmol/L	2.7 (2.6–3.3)	3.1 (2.7–3.3)	2.8 (2.2–3.3)	3.1 (2.8–3.4)	3.6 (2.8–5.4)	3.6 (3.2–3.9)	4.2 (3.2–4.6)	2.7 (2.4–3.8)	4.6 (3.8–5.2)	4.5 (3.8–5.7)	4.5 (4.1–6.1)	4.6 (3.9–6.1)
Hct, %	46 (41–48)	46 (43–48)	46 (43–46)	48 (44–50)	46 (41–49)	49 (42–51)	30 (27–32)	26 (25–28)	28 (26–30)	28 (26–31)	28 (27–28)	29 (27–30)
BE, mEq/L	−3 (−3– −1)	−3 (−4–-2)	−2 (−4– −1)	−3 (−4– −2)	−5 (−8– −2)	−4 (−4– −1)	−1 (−3–0)	1 (−2–2)	−1 (−3–0)	−3 (−4– −1)	−2 (−4–1)	−2 (−3–1)
15 min after resuscitation												
∆MAP, mmHg	−7 (−19 – −5)	−18 (−36 – −6)	−4 (−8 – −1)	0 (−8–6)*	−2 (−13–5)	−2 (−9–7)	ns	−7 (−8–0)	4 (1–6)	5 (−3–9)	8 (1–26)*	2 (−4–14)
∆PPV, %	−1 (−6–7)	4 (0–6)	3 (0–6)	1 (−10–3)	−4 (−7–6)	−4 (−6–1)	ns	0 (−3–3)	−4 (−11–2)	−8 (−16 – −2)	−11 (−13– −7)*	−7 (−9 – −2)
∆ScvO_2_, %	−9 (−14 – −1)	1 (−10–7)	−1 (−7–8)	−6 (−13–5)	6 (−15–15)	8 (−1–10)	ns	0 (−4–8)	9 (0–12)	9 (1–19)	13 (8–24)	19 (−1–26)
∆Lactate, mmol/L	0.3 (−0.3–0.6)	−0.2 (−0.5–0.3)	−0.4 (−1.1–0)	−0.7 (−1– −0.3)	−1.1 (−1.9 – −0.6)*	−1.4 (−1.8 – −1.0)*	ns	−0.1 (−0.7–0.2)	−1.0 (−1.6– −0.6)	−1.7 (−2.2– −0.9)	−1.9 (−2.9– −1.2)*	−2.4 (−2.8– −1.1)*
∆Hct, %	0 (−1–1)	−3 (−3 – −2)	−4 (−5 – −3)	−5 (−7– −5)*	−6 (−10–0)	−7 (−10 – −3)	ns	−2 (−3 – −1)	−6 (−8 – −5)	−9 (−11– −6)	−11 (−12– −10)*	−12 (−14 – −11)*
∆BE, mEq/L	−3 (−3 – −1)	−1 (−2– −1)	1 (0–1)	2 (1–2)*	3 (2–5)*	3 (2–3)*	ns	0 (−1–1)	2 (1–4)	4 (2–5)*	4 (2–7)*	4 (2–5)*
60 min after resuscitation												
∆MAP, mmHg	−19 (−27–0)	−23 (−53– −16)	−14 (−23– −4)	−14 (−31–0)	−21 (−34 – −6)	−11 (−26– −8)	ns	−8 (−11–2)	4 (−3–8)	3 (−3–8)	10 (2–14)*	5 (−5–8)
∆PPV, %	1 (−3–6)	4 (−1–7)	2 (−2–5)	0 (−5–5)	−2 (−11–5)	−3 (−8–3)	ns	0 (−4–6)	−1 (−15–7)	−5 (−10–4)	−11 (−14– −6)*	−7 (−17– −3)
∆ScvO_2_, %	−19 (−27–12)	−14 (−16– −10)	−10 (−13 – −3)	−16 (−24 – −6)	−6 (−20–7)	−8 (−13–4)	ns	−1 (−13–2)	3 (−7–7)	4 (2–6)	10 (4–18)*	19 (−2–21)*
∆Lactate, mmol/L	1.3 (0.4–2.7)	0.7 (0.3–3.0)	−0.2 (−0.3–1.2)	0.8 (−0.1–1.3)	0 (−0.4–0.6)	−0.8 (−1.0–0)*	ns	0.5 (−0.6–1.8)	0.3 (−0.4–1.5)	−0.5 (−0.9–0.8)	−0.4 (−1.4–0.8)	−1.3 (−1.8–2.4)
∆Hct, %	−1 (−4–0)	−2 (−4– −2)	−5 (−6– −1)	−6 (−6– −4)	−5 (−9– −1)	−8 (−13– −3)	ns	−5 (−6– −3)	−6 (−8– −4)	−9 (−11– −7)	−11 (−12– −10)*	−12 (−13– −10)*
∆BE, mEq/L	−7 (−8– −5)	−6 (−9– −4)	−3 (−4– −2)	−3 (−4– −2)	−1 (−2–1)*	0 (−1–1)*	ns	−1 (−4–0)	−1 (−4–2)	1 (−2–4)	1 (−2–4)	2 (−4–3)
Urine ml/kg/h	0.4 (0.3–0.5)	0.5 (0.4–07)	0.7 (0.6–0.9)	0.7 (0.5–0.9)	0.5 (0.3–0.8)	1.2 (0.8–1.4)*	ns	0.7 (0.6–0.9)	0.4 (0.3–0.8)	0.8 (0.6–1.4)	1.4 (0.9–1.7)	1.9 (1.6–2.2)*

#### Hemorrhage

At the start of resuscitation, lactate and PPV had increased, and base excess, MAP, ScvO_2_, and hematocrit had decreased compared to baseline values (*P* < 0.001 for all). At 15 min after resuscitation, animals resuscitated with 75 and 100 ml/kg decreased in hematocrit (*P* < 0.001 for both groups) and lactate (*P* = 0.013 and *P* = 0.014, respectively) and increased in base excess (*P* = 0.002 for both groups) compared to the group receiving 10 ml/kg. At 60 min after resuscitation hematocrit decreased and ScvO_2_ increased in the group receiving 100 ml/kg compared to the group receiving 10 ml/kg (*P* = 0.007 and *P* = 0.021, respectively). The urine production was higher in animals resuscitated with a dose of 100 ml/kg compared to those resuscitated with a dose of 10 ml/kg (*P* = 0.018). Arterial pH and pCO_2_ did not differ between the groups at any of the time points (data not shown).

### Plasma volumes

#### Sepsis

**Fig. 4:**
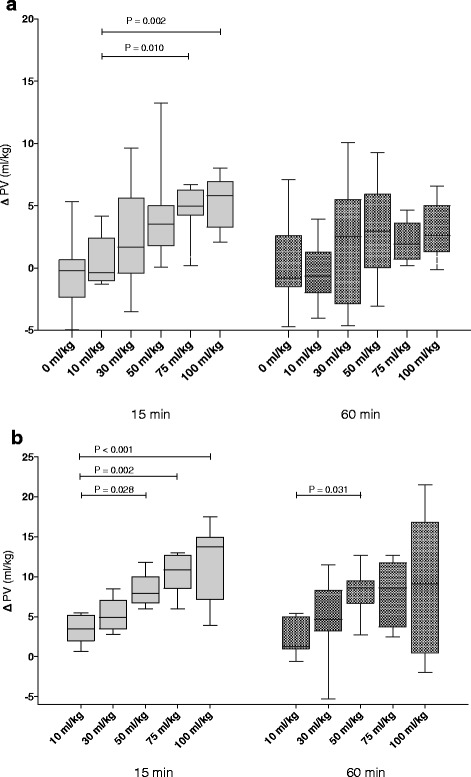
Change in plasma volume (∆ PV) in the sepsis and the hemorrhage groups at 15- and at 60 min after completion of fluid resuscitation. Panel **a**: Change in plasma volume in sepsis groups resuscitated with 10 ml/kg (*n* = 9, second bar from the left at each of the time points), 30 ml/kg (*n* = 10), 50 ml/kg (*n* = 10), 75 ml/kg (*n* = 8), 100 ml/kg (n = 8) and in animals not receiving fluid resuscitation (*n* = 10, first bar form the left, at each of the time points). **b** panel: Change in plasma volumes in hemorrhage groups resuscitated with 10 ml/kg (*n* = 7), 30 ml/kg (*n* = 8), 50 ml/kg (*n* = 8), 75 ml/kg (*n* = 8) and 100 ml/kg (*n* = 8) (* = *P* < 0.05). Data analyzed using one-way ANOVA on ranks and multiple comparisons were adjusted using the Dunn’s method

The baseline plasma volume in the sepsis group was 42.1 (39.5–46.4) ml/kg and decreased to 30.8 (28.1–33.7) ml/kg 4 h after the CLP procedure (*P* < 0.001). Corresponding blood volumes were 72.4 (68.2–79.5) ml/kg and 56.7 (52.6–60.4) ml/kg, respectively. At 15 min after resuscitation, there was a dose-dependent increase in the plasma volume (Fig. 4a). Calculated blood volume increased to at most 54.8 (52.5–57.7) ml/kg at a dose of 100 ml/kg. At 60 min after resuscitation, no differences in the plasma volume change could be detected (*P* = 0.17 for ANOVA).

#### Hemorrhage

The baseline plasma volume in the hemorrhage group was 41.9 (39.8–43.5) ml/kg and decreased to 30.6 (28.7–32.5) ml/kg 2.5 h after the hemorrhage (*P* < 0.001). The corresponding blood volumes were 71.1 (69.1–74.7) ml/kg and 42.5 (39.39–44.8) ml/kg, respectively. Volume of hemorrhage therefore corresponds to a 40% hemorrhage. At 15 min after resuscitation, the plasma volume increased dose-dependently (Fig. 4b). Blood volume at 15 min after resuscitation increased to at most 49.6 (45.3–56.4) ml/kg at a dose of 100 ml/kg. At 60 min after resuscitation, the 50 ml/kg group had increased in plasma volume compared to the 10 ml/kg group, whereas the 75 ml/kg and 100 ml/kg groups did not differ from the 10 ml/kg group (Fig. 4b).

#### Sepsis versus hemorrhage

Plasma volume expansion as a percentage of the administered dose was assessed to test the hypothesis that efficacy of crystalloids as plasma volume expanders differs between the two inflammatory conditions. Average efficacy both at 15 and 60 min was lower in sepsis than in hemorrhage (15 min; 5.9 (2.5–8.8) % vs. 14.5 (12.1–20.0) %, *P* < 0.001, 60 min; 2.9 ([−2.9]-8.3) % vs. 13.3 (8.3–19.0) %, *P* < 0.001, Mann–Whitney test). In sepsis, average efficacy decreased from 15 to 60 min (*P* = 0.006, Wilcoxon matched-pair signed ranks test), whereas it did not differ between the different time points in hemorrhage (*P* = 0.108).

### Plasma oncotic pressure

**Fig. 5:**
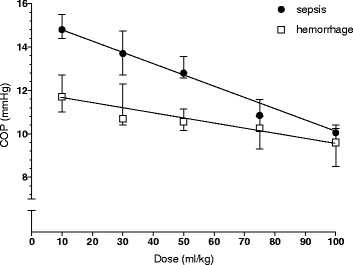
Plasma oncotic pressure at 60 min after completion of resuscitation. Differences in dose-dependent decrease in plasma oncotic pressure between the two groups were analyzed by comparing the slope of the dose–response curves as calculated by linear regression. (*P* < 0.001 for the difference in slope using linear regression with an interaction term)

In the sepsis and hemorrhage groups resuscitated with 10 ml/kg plasma oncotic pressures at 60 min after resuscitation were 14.8 (14.4–15.5) mmHg and 11.7 (11.0–12.7) mmHg, respectively, (Fig. 5). There was a dose-dependent decrease in colloid osmotic pressure in both conditions, which was more marked in sepsis than in hemorrhage (*P* < 0.001 for the difference in slope using linear regression with an interaction term). In sepsis, the plasma oncotic pressure was 10.1 (9.8–10.3) mmHg in the group resuscitated with a dose of 100 ml/kg. This corresponds to a 32% lower plasma oncotic pressure than in the 10 ml/kg group. At this time point, the plasma volume in animals resuscitated with 100 ml/kg had increased by about 10% relative to the plasma volume change observed in animals resuscitated with 10 ml/kg. In hemorrhage, the plasma oncotic pressure was 9.6 (8.5–10.4) mmHg in the group resuscitated with a dose of 100 ml/kg. This corresponds to a 20% lower plasma oncotic pressure than in the 10 ml/kg group. At this time point, the plasma volume in animals resuscitated with 100 ml/kg had increased by about 23% relative to the plasma volume change observed in animals resuscitated with 10 ml/kg.

### Tissue water content

**Fig. 6:**
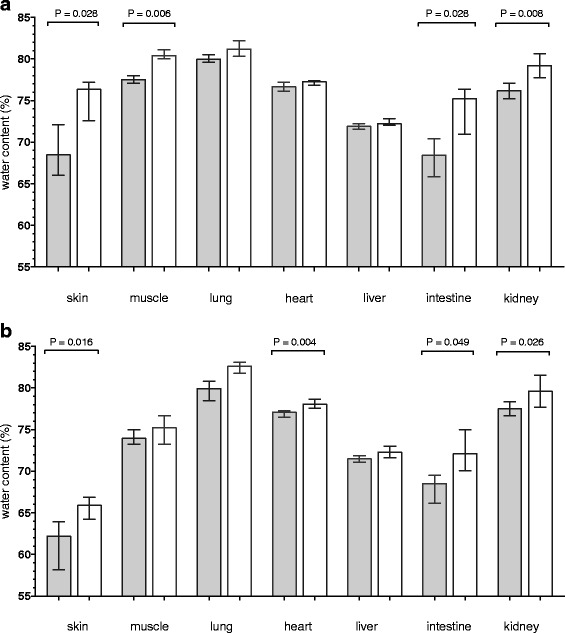
Tissue water content in animals exposed to sepsis (gray bar in panel **a**) or hemorrhage before resuscitation (gray bar in panel **b**, *n* = 4 in both conditions) and after resuscitation with Ringers acetate at a dose of 100 ml/kg (white bars, *n* = 8 in both conditions). Differences in water content were analyzed using Mann–Whitney nonparametric test

The tissue water content in skin, intestine, muscle and kidney was higher in resuscitated group than in the control group in sepsis (Fig. 6). The tissue water content in the skin, intestine, heart, and kidney was higher in the resuscitated group than in the control group in hemorrhage (Fig. 6). In both conditions water content appeared to increase the most in skin and intestines.

## Discussion

Our results show that resuscitation with Ringers acetate in inflammation induced by abdominal sepsis increases plasma volume by about 7% of the infused dose early after resuscitation. In inflammation induced by hemorrhagic shock, Ringers acetate result in an increase in plasma volume by about 18% of the infused volume early after resuscitation. Calculated blood volumes after resuscitation did not reach baseline values even at the highest dose in any of the conditions. Resuscitation with Ringers acetate was associated with a dose-dependent decrease in the plasma oncotic pressure, which was more marked in abdominal sepsis.

Measurement of plasma volume using radiolabeled albumin is considered to be a golden standard and both baseline values and values after CLP in the present study are very similar to those reported in previous studies supporting the reliability of the methodology [14, 19, 20]. Sources of error, such as changes in the rate of extravasation of albumin secondary to increases in permeability, and amount of free iodine has been analyzed in detail previously [14] and it has been concluded that these sources of error are small during the present experimental conditions. Furthermore, our finding of reciprocal changes in hematocrit also supports the reliability of the plasma volume measurements.

Our finding of hypovolemia, increased lactate and a decreased MAP after CLP are in keeping with previous results and support that the model initiates an inflammatory response which mimics many features of human sepsis [14, 21]. Observational studies have shown that it is not uncommon that patients with septic shock receive more than 125 ml/kg during the first hours of resuscitation [22] suggesting that the investigated doses are similar to those used in clinical practice. Also, hemorrhagic shock is suggested to initiate an inflammatory response, which shares many aspects of septic shock despite the differing etiologies [23] and our previous result showing increased levels of TNF-α before resuscitation using an identical protocol support the development of a shock induced inflammatory response in our model [18].

To our knowledge, this is the first study investigating the dose–response relationship of Ringers acetate as a volume expander. Our finding that Ringers acetate expands the plasma volume by about 7% of the infused volume early after resuscitation in sepsis is in agreement with previous studies evaluating effects of a single dose on plasma volume in experimental and clinical inflammation [13–15]. The present results extend the previous findings and indicate that potency appears to be similar across a wide range of doses immediately after resuscitation. However, 1 h after completion of resuscitation no difference in plasma volumes between the different doses could be detected. While this in part can be referred to low statistical power, it should also be noted that mortality appeared to be higher in animals receiving no resuscitation or the lower doses of Ringers acetate. Assuming that hypovolemia contributes to mortality, it is possible that this biased selection of animals available for plasma volume measurement in favor of those with less hypovolemia and/or inflammatory response. Such a bias may have overestimated potency of Ringers acetate in these groups.

Plasma volume expansion by 0.9% saline and Ringer acetate is often assumed to be equal and guidelines do not specify the type of crystalloid to use [1, 2] and, while studies comparing the two are sparse, a study on healthy humans has suggested that 0.9% saline is more potent than Ringers acetate [24]. In contrast, a previous study from our laboratory [13] using similar sepsis and hemorrhage protocols found that a single dose of 0.9% saline (32 ml/kg) increased plasma volume by 0.6 and 20% of the injected dose (corresponding results for Ringers acetate in the present study was 8 and 17%) suggesting that in rat sepsis, Ringers acetate is at least, equally potent as 0.9% saline. Future studies will have to address the relative potency of Ringers acetate and 0.9% saline in humans suffering from inflammatory conditions.

The finding that average efficacy was lower at 1 h align with a clinical study showing that crystalloid induced increases in cardiac output are transient and return to pre-infusion levels within 1–2 h [25]. The previous finding that immediate plasma volume expansion in less severe sepsis is about 20–30% of the infused volume contrasts to our results and suggests that efficacy in sepsis is not uniformly low and could be dependent on the severity of the inflammatory response [26, 27].

In hemorrhagic shock, Ringers acetate was a more potent plasma volume expander than in sepsis. Moreover, no decrease in efficacy during the first hour after resuscitation could be detected. The observation that normovolemia was not restored even at the highest dose of Ringers acetate, align with our previous finding that normovolemia was not achieved even at a dose of 135 ml/kg at 2 h after a hemorrhage of the same magnitude [18]. This contrasts to less severe hemorrhage, in which immediate resuscitation with crystalloids at a dose of about 4.5 the bleed volume restores normovolemia [14, 28]. Taken together our results suggest that normovolemia is very difficult to achieve using Ringers acetate in these two models of inflammation. As mentioned above, in clinical practice patients with sepsis may receive large amounts of crystalloids to correct hemodynamics. While we acknowledge that unresponsiveness to fluid resuscitation in sepsis is likely to be multifactorial our results indicate that poor plasma volume expansion by crystalloids is a potential contributing factor.

Our finding that water content increased mainly in the intestines and skin after resuscitation supports that excess fluid is not uniformly distributed in different tissues and organs but is preferably distributed to organs with a high interstitial compliance [29]. While we did not measure interstitial and intravascular extracellular volumes changes after resuscitation previous studies suggest in experimental hemorrhagic shock the fluid is mainly distributed interstitially [30]. Of note is that no increase in tissue water content in the lungs could be detected after resuscitation. This finding contrasts to previous studies and could be related to lack of power [30].

The question may be asked how the lower efficacy of Ringers acetate as a plasma volume expander in sepsis than in hemorrhage may be explained? The vascular endothelium of all tissues except the brain is freely permeable to electrolytes already during normal conditions it is therefore highly unlikely that an inflammation induced increase in endothelial permeability for electrolytes could directly influence efficacy of crystalloids as plasma volume expanders per-se. However, hypovolemia in sepsis is secondary to redistribution of fluid from the intra- to the extravascular compartment in part due to increased permeability for colloids. This is in contrast to the hypovolemia in hemorrhage, which will initiate compensatory mechanisms to redistribute fluid in the opposite direction in an attempt to maintain normovolemia. Assuming that a crystalloid initially distributes in the extravascular space, the change in the ratio of intravascular to extravascular extracellular volumes is likely to contribute to the low potency of crystalloids in sepsis. Moreover, inflammation blunts homeostatic mechanisms that contribute to the plasma volume expansion observed in non-inflammatory conditions and by this mechanism contribute to the lower potency. Also inflammation-induced changes in the structure of the interstitial matrix may contribute to the low potency and duration of effect of crystalloids in severe systemic inflammation [31].

The result that plasma oncotic pressure decreased dose-dependently after hemorrhagic shock can be explained by the dilution of plasma proteins by Ringers acetate. This is in contrast to sepsis, in which, the decrease in plasma oncotic pressure was two times higher than what can be explained by dilution alone. This result suggests that Ringers acetate induce a dose-dependent increase in extravasation of albumin and/or a decrease in lymphatic return of albumin. At least two mechanisms may contribute to an increased extravasation of albumin in sepsis. Firstly, the increased water content in the interstitium prior to resuscitation secondary to vascular leak may have increased the volume of the distribution for albumin in the interstitium. This may have caused increased diffusion of albumin from the vessels to the interstitium. Secondly, increased permeability in sepsis is suggested to be secondary to an increased number of large pores [32] and/or to increased functional pore size due degradation of the glycocalyx [17]. Given that convective transport of albumin occurs through these pores, it is possible that convective transport of albumin due to a transiently increased transcapillary hydrostatic pressure during the distribution of crystalloids is larger in sepsis. Whatever the mechanism, this finding represents a previously unrecognized side-effect of crystalloid resuscitation in sepsis that is likely to contribute both to relatively poor potency and short duration of crystalloids as plasma volume expanders in sepsis.

### Limitations

We acknowledge that our models suffer from several limitations. Most importantly, no empiric antibiotic therapy was initiated. Also vasoconstrictors and inotropes were not used in the sepsis model. We also acknowledge that a 40% hemorrhage would not normally be resuscitated using only crystalloids. Furthermore, the short observation time after resuscitation does not allow us to draw any conclusions about a more long-term dose-dependent effect on hemodynamics and more importantly on patient-important outcomes.

For standardization purposes all groups were resuscitated in 30 min and consequently flow rates of resuscitation fluids varied between the groups. While we cannot exclude this may have influenced our result, previous data indicate that flow rate does not influence plasma volume expansion by crystalloids [13].

## Conclusions

We conclude that the efficacy of Ringers acetate as a plasma volume expander is context dependent. Plasma volume expansion is lower than previously realized across a wide range of doses and normovolemia may be impossible to achieve in sepsis. Resuscitation with Ringers acetate in sepsis induces a dose-dependent decrease in plasma oncotic pressure, which cannot be explained by dilution of plasma proteins only.

## Abbreviations

ANOVA: Analysis of variance
CI: Confidence interval
CLP: Cecal ligation and puncture
IQR: Interquartile range
MAP: Mean arterial pressure
PP: Pulse pressure
PPV: Pulse pressure variation
PV: Plasma volume
